# Activation of μ-opioid receptors inhibits calcium-currents in the vestibular afferent neurons of the rat through a cAMP dependent mechanism

**DOI:** 10.3389/fncel.2014.00090

**Published:** 2014-03-27

**Authors:** Emmanuel Seseña, Rosario Vega, Enrique Soto

**Affiliations:** Instituto de Fisiología, Universidad Autónoma de PueblaPuebla, México

**Keywords:** neurotransmitter, synapse, inner ear, auditory, hair cells, efferent system, neuromodulation

## Abstract

Opioid receptors are expressed in the vestibular endorgans (afferent neurons and hair cells) and are activated by the efferent system, which modulates the discharge of action potentials in vestibular afferent neurons (VANs). In mammals, VANs mainly express the μ opioid-receptor, but the function of this receptors activation and the cellular mechanisms by which they exert their actions in these neurons are poorly studied. To determine the actions of μ opioid receptor (MOR) and cell signaling mechanisms in VANs, we made perforated patch-clamp recordings of VANs that were obtained from postnatal days 7 to 10 (P7–10) rats and then maintained in primary culture. The MOR agonist [D-Ala^2^, N-Me-Phe^4^, Gly^5^-ol]-enkephalin (DAMGO) inhibited the total voltage-gated outward current; this effect was prevented by the perfusion of a Ca^2+^-free extracellular solution. We then studied the voltage-gated calcium current (I_ca_) and found that DAMGO Met-enkephalin or endomorphin-1 inhibited the I_Ca_ in a dose-response fashion. The effects of DAMGO were prevented by the MOR antagonist (CTAP) or by pertussis toxin (PTX). The use of specific calcium channel blockers showed that MOR activation inhibited T-, L- and N-type I_Ca_. The use of various enzyme activators and inhibitors and of cAMP analogs allowed us to demonstrate that the MOR acts through a cAMP dependent signaling mechanism. In current clamp experiments, MOR activation increased the duration and decreased the amplitude of the action potentials and modulated the discharge produced by current injection. Pre-incubation with PTX occluded MOR activation effect. We conclude that MOR activation inhibits the T-, L- and N-type I_Ca_ through activation of a Gα_i/o_ protein that involves a decrease in AC-cAMP-PKA activity. The modulation of I_Ca_ may have an impact on the synaptic integration, excitability, and neurotransmitter release from VANs.

## Introduction

The μ opioid receptor (MOR) participates in reward-seeking behavior, analgesia, physical dependence, and the modulation of respiratory frequency (Fields, 2011; Raehal et al., 2011). The MOR is a member of the G protein coupled receptors; it is predominantly coupled to a Gα_i/o_ protein, which decreases the adenylyl cyclase activity, thereby lowering the cellular level of cAMP and the PKA activity (Law, 2011). Among other cellular processes affected by opioid receptors, the MOR modulates voltage-dependent calcium, sodium and potassium channels (Witkowski and Sulczyk, 2006; Law, 2011).

Since the discovery of opioid peptide expression in the inner ear by Fex and Altschuler (1981), substantial research has addressed the expression of opioid peptides in the inner ear. Studies in the vestibular system showed that opioid peptides and their receptors are expressed both at the central and at the peripheral levels. In the vestibular efferent neurons of the dorsal group of the gerbil brainstem, the expression of Met-enkephalin and enkephalin precursors was demonstrated (Perachio and Kevetter, 1989; Ryan et al., 1991). In the vestibular nuclei of dogs, the immunoreactivity to Met-enkephalin has been identified (Pego-Reigosa et al., 2000), while preproenkephalin was found in the magnocellular vestibular nucleus in salmon (Vecino et al., 1995). Recordings in slices obtained from the medial vestibular nucleus (MVN) of the rat have shown that delta-opioid receptor agonists decreased the neuronal tonic discharge (Sulaiman and Dutia, 1998). Further, the use of preproenkephalin antisense and naloxone significantly delayed vestibular compensation (Kitahara et al., 2006).

In the vestibular endorgans, the MOR was found in the afferent neuron terminals (Popper et al., 2004). In recordings of the frog vestibular afferent nerve, the local perfusion of enkephalin decreased the basal resting discharge (Andrianov and Ryzhova, 1999), whereas in the axolotl vestibule, κ opioid receptor (KOR) activation produced an inhibition of the Ca^2+^ current in hair cells and a postsynaptic facilitation of the afferent response mediated by MOR (Vega and Soto, 2003). Consistent with these basic science results, in patients with vestibular alterations, morphine administration decreased nystagmus, and vertigo produced by rotatory stimulation (Rubin and Winston, 1950), while it increased the spontaneous nystagmus in opioid addicts (Kurnatowski, 1992; Benyamin et al., 2008; Baier et al., 2010). The use of a selective KOR agonist (salvinorin-A) in controlled conditions has been found to produce hallucinations with typical sensations of changes in spatial orientation (Johnson et al., 2011).

The dynamics of the vestibular endorgans depend on the interactions between the efferent system modulation and the output from hair cells and afferent neurons (Cullen, 2012). After unilateral vestibular damage, balance is partially recovered after approximately one month (Beraneck and Idoux, 2012). In this process, the efferent system may contribute to gain readjustment between both labyrinths; opioid peptides, along with other neurotransmitters such as ACh and CGRP, appear to play a fundamental role in readjusting the gain of the system (Perachio and Kevetter, 1989; Ryan et al., 1991; Holt et al., 2011).

Preliminary results from our laboratory showed that MOR activation affects the voltage-gated calcium current (I_Ca_) in the afferent neurons, while other ionic currents are not significantly modified by the use of MOR related drugs. Voltage-gated calcium channels are susceptible to modulation by opioid receptor activation. While a large number of works address the modulation of high voltage activated calcium channels (HVA) (Bourinet et al., 1996; Rusin and Moises, 1998; Connor et al., 1999; Margas et al., 2007; Lemos et al., 2012), few reports have focused on the modulation of the low voltage activated calcium channels (LVA) (Schroeder et al., 1991; Formenti et al., 1995; Yang et al., 2000). The signaling mechanism implicated in the opioid receptor modulation of calcium channels includes voltage-dependent (VD) and voltage-independent (VI) mechanisms (Dascal, 2001). The VD mechanism entails the Gβγ subunit and supports the willing-reluctance mode of the calcium channels. The VI mechanism involves diffusible second messengers, generally cAMP; however, there is increasing evidence that the Gβγ can modulate other second messengers apart from cAMP (Smrcka, 2008).

The aim of this work was to determine the opioid receptor function, the cell signaling mechanisms of MOR modulation and the type of I_Ca_ modulated by MOR in the vestibular primary afferent neurons.

## Materials and methods

Animal care and procedures were in accordance with the APS's Guiding Principles in the Care and Use of Vertebrate Animals in Research and Training and the “Reglamento de la Ley General de Salud en Materia de Investigación para la Salud of the Secretaría de Salud de México.” All efforts were made to minimize animal suffering and to reduce the number of animals used, as outlined in the “Guide for the Care and Use of Laboratory Animals” issued by the National Academy of Sciences. Animals were supplied by the “Claude Bernard” animal house of the Autonomous University of Puebla.

### Primary culture of vestibular afferent neurons (VAN)

Long-Evans rats from postnatal days 7 to 10 (P7–10) without sex distinction were used for the experiments. Animals were anesthetized using sevoflurane and euthanized by decapitation. The head was cleaned rigorously with 75% ethanol. The inferior maxilla was removed, and the upper part of the skull and the brain were removed. The vestibular ganglia were identified, dissected and placed in Leibovitz medium (L-15) to proceed with enzymatic and mechanical dissociation (Soto et al., 2002). To dissociate the ganglia, we used 1.25 mg/ml trypsin and 1.25 mg/ml collagenase type IA dissolved in L-15 medium. A fire-polished Pasteur pipette was used to homogenize the tissue; the homogenate was then centrifuged at 2500 × g for 5 min. The supernatant was discarded, and the procedure was repeated two additional times. The isolated ganglia neurons were plated on cover slides pretreated with 100 μg/mL poly-D-lysine (Sigma–Aldrich, St. Louis, MO) in 35-mm Petri dishes (Corning, Lowell, MA, USA), with 2 mL of modified L-15 medium (supplemented with 10% FBS, 1000 IU penicillin, 15.7 mM NaHCO_3_, 15.8 mM HEPES, and the pH adjusted to 7.7 with NaOH). The cells were maintained in a 95% air 5% CO_2_ atmosphere, in a humidified incubator at 37°C for 18–24 h until the electrophysiological record. To study whether the responses observed in P7–10 day old rats were also present in older rats, an experimental series was also performed in P28–30 day old rats.

### Solutions

To record the I_Ca_, we used an extracellular solution with the following composition (in mM): 130 TEA-Cl, 10 4-AP, 5 CsCl, 2.5 CaCl_2_, 10 HEPES and 10 glucose; the pH was adjusted to 7.4 with HCl. The pipette solution had (in mM): 130 CsCl, 10 TEA-Cl, 10 EGTA, 15 HEPES and 0.134 CaCl_2_; the pH was adjusted to 7.2 with CsOH. For the current clamp experiments and for the recording of the voltage-gated total current, we used a normal extracellular solution with the following composition (in mM): 140 sodium isothianate, 5.4 potassium gluconate, 1.8 CaCl_2_, 1.2 MgCl_2_, 10 HEPES and 10 glucose; the pH was adjusted to 7.4 with NaOH. The pipette was filled with the following solution (in mM): 125 potassium gluconate, 12 sodium isothianate, 0.134 CaCl_2_, 10 EGTA, and 10 HEPES; the pH was adjusted to 7.2 with KOH. The osmolarity of the solutions was ~300 mOsm. In the intracellular solutions Amphotericin B (300 μM) or nystatin (260 μM) both from Sigma-Aldrich, Co. (St. Louis, MO, USA) were dissolved in 2 mM DMSO before the recordings and added to the pipette solutions freshly. Solutions were protected from light to prevent ionophore degradation. With this procedure time to reach an input resistance ≅ 20 MΩ was about 25 min after establishing the gigaseal.

### Drugs

The drug application was made by using a gravity-driven flow system (flow rate of approximately 0.5 mL/s) consisting of three square perfusion tubes coupled to a step motor and an electronically controlled valve system (SF-77B; Warner Instruments, Hamden, CT, USA). The drugs used were the MOR agonists [D-Ala^2^, N-Me-Phe^4^, Gly^5^-ol]-enkephalin (DAMGO), Met-enkephalin and endomorphin-1; the MOR antagonist H-D-Cys-Tyr-D-Trp-Arg-Thr-Pen-Thr-NH_2_ (CTAP); the Gα_i/o_ protein inhibitor pertussis toxin of *Bordetella perussis* (PTX); the adenylyl cyclase activator forskolin; the cAMP analog 8-Br-cAMP; the phosphodiesterase inhibitor IBMX; the PKA inhibitor H-89; the phosphatase inhibitor okadaic acid; the PKC inhibitor NPC15437; the PKC activator porbol-myristate-acetate (PMA); the N type channel blocker ω-ctx-GVIA; the L type channel blocker nifedipine; and the T type channel blocker NiCl_2_. The NPC15437 was acquired from Research Biochemical International (Natick, MA, USA), the okadaic acid from Alomone Labs (Jerusalem, Israel), and the remaining drugs were from Sigma-Aldrich Co. (St. Louis, MO, USA). For stock solutions, the hydro soluble drugs were diluted in deionized water, and the non-hydro soluble drugs were diluted in DMSO (0.2% DMSO final concentration). The ω-ctx-GVIA solution also contained 0.1 mg/ml cytochrome C (Sigma) to saturate unspecific binding sites.

### Electrophysiological recording

Membrane ionic currents and cell-voltage responses were studied using the perforated-patch voltage-clamp and current-clamp technique. Experiments were performed at room temperature (23–25°C). Ionic currents were recorded using an Axopatch 200 B amplifier (Molecular Devices, Union City, CA, USA). Command-pulse generation and data sampling were controlled by the pClamp-10 software (Molecular Devices) using a 16-bit data acquisition system Digidata 1440 A (Molecular Devices). Data were sampled at 20 kHz; the low-pass filter was set at 10 KHz to evaluate the passive properties and at 2 KHz for the current recordings. The passive properties of the cells (membrane capacitance C_m_, membrane resistance R_m_, access resistance R_a_ and time constant τ) were measured on-line with the pClamp program with a pulse to 5 mV from a holding of −70 mV. The series resistance was electronically compensated to 80%. The recording electrodes were made from borosilicate-glass capillaries 1.2 mm (TW 120, WPI, Sarasota, FL, USA) with resistances between 1.5 and 3 MΩ once filled with pipette solution. The recording chamber was continuously perfused with the corresponding extracellular solution.

To create the I-V relationship, we used a protocol of voltage pulses that ranged from −100 to 50 mV, with 200 ms durations and steps of 10 mV and 5 s inter sweeps; the holding voltage (*V_H_*) was −100 mV or −40 mV (to elicit the LVA + HVA or the HVA currents) as indicated in each experimental series. To determine the LVA and HVA current in the same sweep, we used a *V*_H_ of −80 mV followed by a pulse to −50 mV of 100 ms and then a pulse to −10 mV with a duration of 100 ms. To evaluate the voltage-dependent modulation of the I_Ca_ by opioid peptides, we used a protocol that started from a *V*_H_ = −80 mV and gave two depolarizing pulses (*P*_1_ and *P*_2_, both to −10 mV, 40 ms) separated by a depolarizing pulse to 80 mV during 40 ms; in consequence, the 80 mV pulse acts as a prepulse of *P*_2_. Thus, if an interaction between the Gβγ complex and calcium channels is present, the current caused by *P*_2_ should be bigger than that caused by *P*_1_, and *P*_2_/*P*_1_ > 1. The time between *P*_1_ and *P*_2_ was 70 or 1065 ms to discard the possibility of an inactivation accumulation of the Ca^2+^ channels.

In the current clamp recordings, to study the action potential (AP) waveform, cells were stimulated with a current pulse injection of 300 pA with a duration of 3 ms, followed by 10 ms of zero current and a hyperpolarizing pulse of 200 pA with a duration of 500 ms (to produce a rebound potential). To evaluate the repetitive AP discharge of the afferent neurons, a pulse of 400 pA and 500 ms in duration was used. Cells were also subjected to sinusoidal stimulation (5 Hz and amplitude from 0.2 to 1 nA); the stimulus amplitude was adjusted in order for the neurons to have an unsaturated response (1 to 1 phase looking between the stimulus and AP discharge were considered as saturating). Instead, a stimulus amplitude in which cells discharge in some stimulus cycles and not in other cycles (ideally 50%) was chosen. This allows the cell to increase or decrease its AP discharge when experimentally manipulated (i.e., the use of drugs).

### Data analysis

Recordings were analyzed off-line using Clampfit 10.2 (Molecular Devices) and Sigma Plot 8.0 (Systat Software, Richmond, CA, USA). To construct the I-V relationship, the current amplitude was measured at the peak current in each voltage and the voltage error produced by uncompensated series resistance was corrected (R_ca_ was 11 ± 1 mΩ). Leak subtraction protocols were not used, instead a linear fit to the current at voltages between −100 to −70 mV was subtracted from the IV relationship. The junction potential calculated using the Henderson-Hasselbalch equation was 4.3 mV and was compensated. The current data are presented as the current density (pA/pF). The conductance (*g*) was obtained in two ways: (i) it was calculated from the equation *g* = I_P_ / (V_r_ − V_e_), where I_P_: peak current, V_r_: recording voltage, and V_e_: equilibrium voltage (obtained from experimental measurements), (ii) it was directly measured from tail currents elicited with a protocol that provided a voltage pulse from −100 to 50 mV (40 ms duration, Δ inter-steps of 10 mV and time inter- sweeps 10 s) from a *V*_H_= −80 mV; the tails were normalized with the tail value at 30 mV. The conductance data were fitted with a simple Boltzmann equation of the form *f*(*x*) = [(A_1_−A_2_)/(1 + exp (V − V_1/2_ /*S*))] + A_2_, where A_1_: initial value, A_2_: final value, *S*: Boltzmann constant, V_1/2_: voltage at which 50% of the current is activated and V: test voltage. For the LVA conductance, the data between −100 and −40 mV were fitted; for the HVA conductance, the values between −100 and 40 mV were fitted omitting the values from −70 to −40 mV.

For the analysis of the AP waveform, the phase diagrams were plotted (V_m_ vs. dV_m_/d_t_) and measurements made from these diagrams. The parameters measured included: the V_m_ (membrane voltage at zero current injection), the amplitude (from V_m_ to the AP peak), the threshold (value at which dV_m_/dt changes suddenly), the duration at 50% of the amplitude, the maximum depolarization rate (MDR), the maximum repolarization rate (MRR) and the post-hyperpolarization amplitude (PHP). For the pulse-evoked repetitive discharge, we counted the APs produced by the depolarizing current injection.

In experiments in which drugs were applied, a single drug concentration was tested for each cell to assure independency of the data sampling. For the statistical analyses, a paired Student's *t*-test was used in cases in which the dependent variable was measured before and after treatment. In the cases in which more than one treatment was used, a One-Way ANOVA with a Tukey *post-hoc* test was used. The significance level was established as α = 0.05. Data were considered significantly different at *P* = 0.05. The results are shown as the means ± s.e.m.

## Results

### Currents modulated by MOR activation

**Figure 1:**
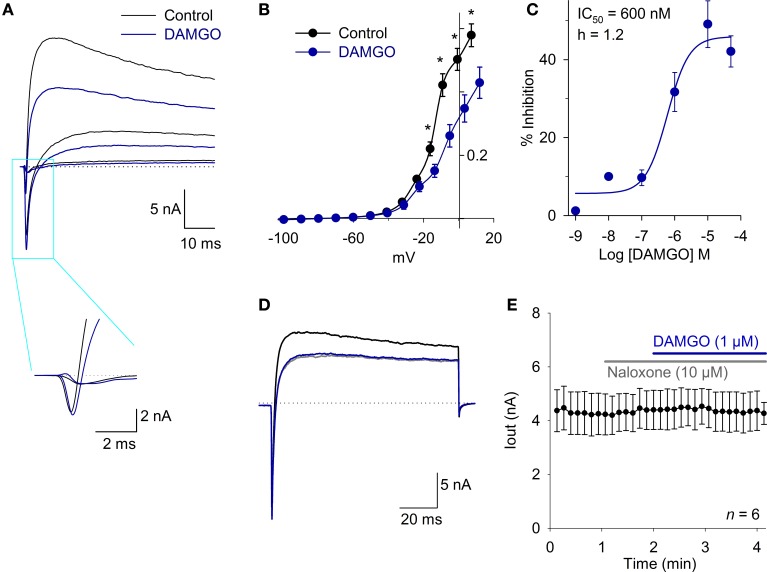
**The MOR activation inhibits an outward current in whole cell current recording. (A)** Traces of whole cell total current showing inward and outward currents under control conditions and after 1 μM DAMGO perfusion, the fast inward current (mainly attributable to Na^+^) did not change, but the outward current (attributable to K^+^) decreased significantly. Inset shows the fast inward current in a time expanded scale. The dotted lines indicate zero current. **(B)** The current-voltage relationship for the outward current showed a significant effect of DAMGO which decreased the maximum current 32% at 20 mV. **(C)** Concentration response relationship for the DAMGO inhibitory effect on the outward current was fitted with a Hill equation with a CI_50_ = 600 nM and Hille coefficient of 1.2. **(D)** Voltage clamp recordings of whole cell current under control condition (black trace). The perfusion with a Ca^2+^ free extracellular solution significantly decreased the outward current (gray trace), and the effect of 1 μM DAMGO was completely occluded by perfusion with a Ca^2+^ free solution (blue trace). The dotted lines indicate zero current. **(E)** Time course of outward current amplitude in control, after 10 μM naloxone perfusion, which exerted no effect, but completely occluded the action of 1 μM DAMGO, indicating that DAMGO effect is specifically mediated by opioid receptor activation. ^*^Indicates a significant difference *P* < 0.05.

To evaluate whether MOR activation produced significant modification of the ionic currents in the VANs, the total current was registered using a protocol from a *V*_H_ = −70 mV, pulses from −130 to 50 mV in steps of 10 mV and a duration of 200 ms every 10 s. The inward current (attributable to Na^+^ current) was not modified by DAMGO in a concentration ranging from 1 nM to 10 μM (*n* = 25, *P* > 0.05). The inward current density at the IV relationship peak was 385 ± 55 pA/pF under control conditions and 323 ± 49 pA/pF after the application of 1 μM DAMGO (*n* = 6, *P* = 0.80) (Figure 1A). The outward K^+^ current decreased with the DAMGO application. At a potential of 20 mV, the current diminished from 220 ± 22 pA/pF in controls to 150 ± 20 pA/pF with 1 μM DAMGO (*n* = 7, *P* < 0.05) (Figures 1A,B). The DAMGO effect in the outward current was fitted with a concentration dependent curve with IC_50_ = 600 ± 360 nM (*n* = 26) (Figure 1C). The DAMGO effect was completely occluded by naloxone (control current was 192 ± 48 pA/pF vs. naloxone and DAMGO 195 ± 47 pA/pF, *n* = 5, *P* = 0.97) and also by perfusion of a calcium free extracellular solution (control current was 164 ± 17 pA/pF vs. 161 ± 17 pA/pF after Ca^2+^ plus DAMGO, *n* =10, *P* = 0.99) (Figures 1D,E). This initial approach indicated that the calcium-dependent potassium current (I_K,Ca_) amplitude decreased as a consequence of the inhibition of the I_Ca_. For this reason, we decided to study the modulation of the voltage-gate calcium current by MOR activation.

### Calcium current characteristics in the VANs

**Figure 2:**
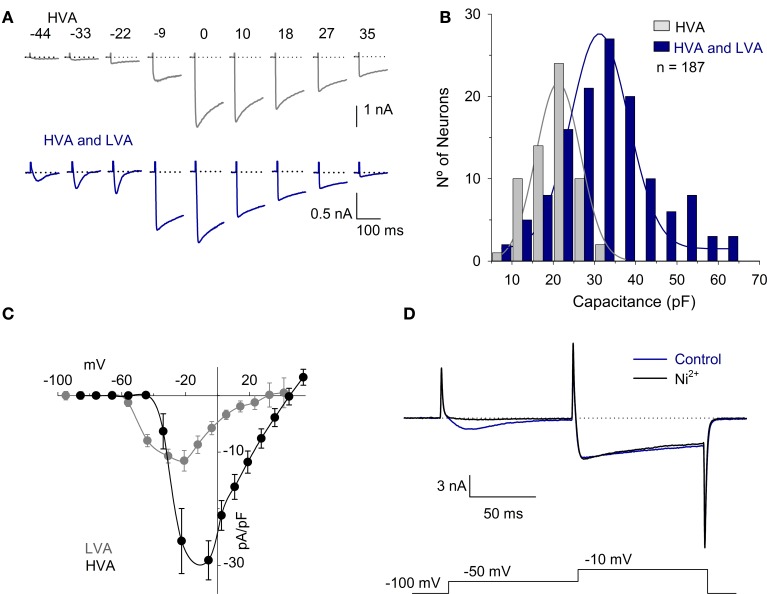
**HVA and LVA calcium currents in vestibular afferent neurons. (A)** Representative traces of the I_Ca_ produced by voltages between −50 mV and 30 mV with a *V*_H_ = −100 mV in neurons with HVA current and in those with both HVA and LVA currents. The dotted lines represent zero current. **(B)** Histogram showing the C_m_ vs. the number of neurons. The neurons with both HVA and LVA I_Ca_ (68%) are distributed across the range of C_m_ from 9 to 62 pF with a mean of 32 ± 1 pF. The neurons with the HVA I_Ca_ are distributed across the range of C_m_ from 10 to 32 pF with a mean of 20 ± 1 pF. Continuous lines were fit with a normal function with R^2^ > 0.95. **(C)** I-V relationship of the LVA I_Ca_ (obtained by subtracting the peak current with *V*_H_= −40 mV from that obtained with a *V*_H_ = −100 mV) and HVA I_Ca_. (measured in cells that expressed the HVA but not the LVA). Because the LVA current is clearly visible at −50 mV with a *V*_H_ = −100 mV and at this voltage the HVA current is not yet activated, this was the voltage selected to study the LVA current. **(D)** Recording of the LVA and HVA components using a double pulse voltage clamp protocol under control conditions and after the use of 100 μM Ni^2+^. The use of Ni^2+^ completely block the LVA component without significantly modifying the HVA.

The I_Ca_ was recorded in 187 neurons using solutions designed for the I_Ca_ recording. The C_m_ of these neurons was 30 ± 1 pF (range 10–62 pF). All neurons had the HVA calcium current, while 68% of the cells (127/187) also had the inactivating LVA calcium current (Figure 2A). The neurons with HVA and LVA currents were typically larger neurons with a C_m_ of 32 ± 1 pF (*n* = 127), while neurons that expressed only the HVA current were significantly smaller in size with a C_m_ of 20 ± 1 pF (*n* = 60; *P* < 0.001) (Figure 2B). The current voltage (I-V) curve of the I_Ca_ was studied using two different holding potentials: a hyperpolarized potential (either −100 or −80 mV as indicated in each case) and a depolarized potential (−40 mV). At both *V*_H_, the maximum current was achieved at a voltage of approximately −10 mV. With the *V*_H_ = −40 mV, the LVA current was not detectable; with the *V*_H_ = −100 mV, the LVA was clearly distinguishable. The LVA current activated at −59 mV (from a *V*_H_ = −80 mV), had a peak value of 12 ± 2 pA/pF at −44 mV and inactivated with a τ of 58 ± 10 ms (*n* = 13) (Figure 2C). Voltage clamp recording at two test pulses showed that 100 nM Ni^2+^ reduce the LVA current 92 ± 4% (*n* = 5, *P* = 0.002) while the reduction of the HVA component was 2 ± 5% (*P* = 0.98) (Figure 2D). Since using a *V*_H_ of −100 mV the LVA current is already activated at −50 mV (−44 mV with Rs and liquid junction potential correction), and at this voltage the HVA current is not yet active, this was the voltage selected for measurements of the LVA current. The HVA current activated at −40 mV, had a peak amplitude at −10 mV and partially inactivated (19 ± 3% of the peak value with a τ of 659 ± 110 ms, *n* = 21).

### MOR modulates both LVA and HVA I_Ca_

**Figure 3:**
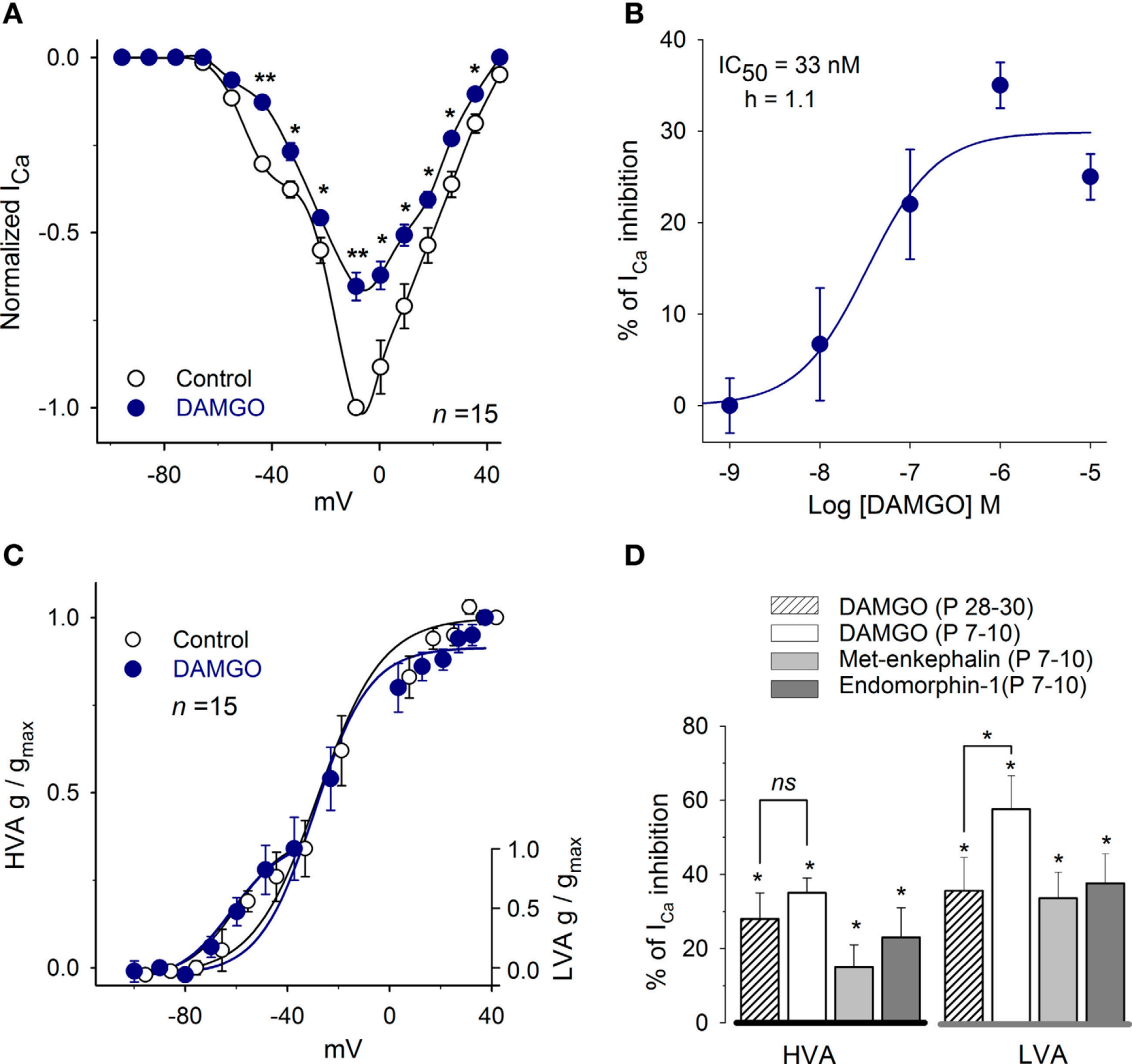
**MOR activation mediates inhibition of the I_Ca_. (A)** Dose-response curve for DAMGO inhibition of the peak I_Ca_ (*n* = 35). **(B)** The control I-V relationship for the I_Ca_ (*V*_H_ = −100 mV) showed a complex form with a hump at −50 mV corresponding to the LVA currentand a peak at approximately −10 mV corresponding to the HVA plus LVA current. The perfusion of 1 μM DAMGO inhibited both components of the I_Ca_. **(C)** The use of 1 μM DAMGO did not significantly modify the parameters of the Boltzmann fit (V_1/2_ and S) to the conductance of the LVA or HVA components. **(D)** Bar graph showing the inhibitory action of 1 μM DAMGO, 1 μM Met-enkephalin and 1 μM endomorphin-1 on the HVA (measured at the peak of the I-V relationship) and the LVA (measured at −50 mV) components. (Statistical significance ^*^P < 0.05, ^**^P < 0.01).

The effects of MOR activation on the I_Ca_ were evaluated using the agonists DAMGO, Met-enkephalin and endomorphin-1. In P7–10 rats, DAMGO was used in concentrations ranging from 100 pM to 10 μM. DAMGO produced a dose-dependent inhibition of the I_Ca_ with an IC_50_ of 33 ± 5 nM and with a Hill coefficient of 1.1 (*n* = 35; Figures 3A,B). The maximum inhibition was attained with 1 μM DAMGO that inhibited 58 ± 7% of the current at −47 mV (LVA) and 35 ± 4% at −14 mV (HVA, *n* = 15, *P* = 0.01); these results indicate that MOR activation inhibits both the LVA and HVA Ca^2+^ current components. Because of these results, the concentration of 1 μM was selected for further testing MOR activation.

Conductance curves showed that DAMGO inhibition of the I_Ca_ did not significantly modify the half activation voltage or the slope of the LVA or HVA current components (LVA: *V*_1/2_ = −55 ± 8 mV, *S* = 7 ± 1 vs. *V*_1/2_ = −54 ± 1 mV, *S* = 7 ± 1. HVA: *V*_1/2_ = −30 ± 3 mV, *S* = 9 ± 1 vs. *V*_1/2_ = −30 ± 3 mV, *S* = 11 ± 1; Figure 3C).

The actions of 1 μM Met-enkephalin and 1 μM endomorphin-1 were also evaluated. Met-enkephalin inhibited the HVA I_Ca_ in 15 ± 6% (*n* = 5, *P* = 0.04) and endomorphin-1 inhibited the I_Ca_ in 23 ± 8% (*n* = 5, *P* = 0.05; Figure 3D). In the LVA, Met-enkephalin inhibited the I_Ca_ in 34 ± 10% (*n* = 5, *P* = 0.07) and endomorphin-1 inhibited the I_Ca_ in 38 ± 8% (*n* = 5, *P* = 0.04; Figure 3D).

### MOR action is preserved during development

To determine whether the modulation of the I_Ca_ by MOR is preserved in older ages, an experimental series using P28–30 rats was performed (this was the only experimental series in which P28–30 rats were used). The perfusion of 1 μM DAMGO in P28–30 rats inhibited the HVA I_Ca_ 28 ± 7% (*n* = 9, *P* = 0.03) and the LVA I_Ca_ 36 ± 9% (*n* = 6, *P* = 0.02, Figure 3D). The inhibition produced by DAMGO at both ages (P7–10 vs. P28–30) was not significantly different for the HVA current (*P* = 0.30) but was significantly lower for the LVA in P28–30 rats (*P* = 0.04); these findings suggest that the LVA current decreased with age or that the LVA current uncouples from the second messenger system in older ages; changes in the MOR expression or sensitivity should in principle be reflected in both the LVA or the HVA current components. Because the I_Ca_ density did not change between the P7–10 and the P28–30 (12 ± 2 pA/pF and 11 ± 1 pA/pF) groups, these results showed that the inhibitory effects of MOR in the calcium current persisted with age, although with small but significant variation in relation to the LVA current.

### HVA I_Ca_ subtypes modulated by MOR activation

**Figure 4:**
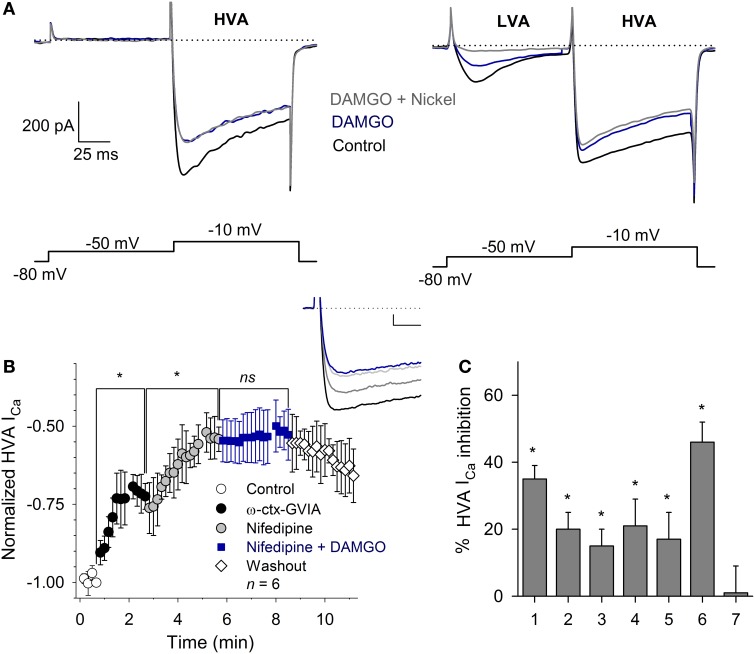
**Calcium current modulation by MOR activation. (A)** Representative traces of the I_Ca_ in neurons with (right) and without (left) LVA current. Two-pulse voltage protocol (shown below) evidenced the LVA and HVA currents. Use of 1 μM DAMGO inhibited both I_Ca_ components. Nickel (100 μM) blocked the LVA current without affecting the HVA component. The dotted line represents zero current. **(B)** Time course of the HVA current in the control and after the application of 3 μM ω-ctx-GVIA, followed by 10 μM nifedipine and nifedipine plus 1 μM DAMGO (*n* = 6; ns, non-significant; ^*^P ≥ 0.5). The application of ω-ctx-GVIA and nifedipine occluded the DAMGO effect. Insert, shows traces of the I_Ca_ in the control condition and with sequential application of ω-ctx-GVIA (gray), nifedipine (light gray) and the co-application of nifedipine and DAMGO (blue). Calibration bars 500 pA, 10 ms. **(C)** Bar graph showing the various experimental conditions used to discern the subtype of Ca^2+^ channels coupled to MOR activation, including: 1- DAMGO, 2- ω-ctx-GVIA, 3- DAMGO + ω-ctx-GVIA, 4- nifedipine, 5- nifedipine + DAMGO, 6- ω-ctx-GVIA + nifedipine, 7- DAMGO + ω-ctx-GVIA + nifedipine. ^*^P ≤ 0.05 with respect to the respective control.

To define the subtypes of Ca^2+^ currents inhibited, we used a voltage clamp protocol with a pulse to −50 mV (with 100 ms duration, to activate the LVA current), followed by a pulse to −10 mV (with 100 ms duration, to activate the HVA current) from a *V*_H_ = −80 mV. With this protocol, the perfusion of 1 μM DAMGO inhibited 60 ± 10% of the LVA current (*n* = 5, *P* = 0.03); the subsequent co-application of 100 μM nickel produced an additional inhibition reaching a total inhibition of 92 ± 4% of the control current (*n* = 5, *P* = 0.02). The inhibition of the HVA current (measured at −10 mV) was 32 ± 6% (*n* = 9, *P* = 0.03), and the co-application of 100 μM Ni^2+^ did not produce additional changes in the HVA current (97 ± 6%, *P* = 0.83 with respect to DAMGO effect). These results suggest that MOR activation partially inhibits both the LVA and HVA components of the I_Ca_ and that 100 μM nickel selectively blocks the LVA I_Ca_ component without affecting the HVA component of the current (Figure 4A).

The HVA component of the I_Ca_ in VANs is formed by the L-, N-, P/Q- and R- calcium channel subtypes (Desmadryl et al., 1997). To study the HVA calcium channel subtypes inhibited by MOR activation, we used specific blockers for each HVA component. The perfusion of the L-type blocker nifedipine (10 μM) decreased the I_Ca_ 21 ± 8% (*n* = 11, *P* = 0.03); the subsequent co-application of 1 μM DAMGO additionally decreased the I_Ca_ 17 ± 8% for a total inhibition of 38 ± 8%, indicating that nifedipine occluded 47% of the DAMGO effect (*n* = 11, *P* = 0.02, ANOVA). The perfusion of the specific N-type calcium channel blocker ω-ctx-GVIA (3 μM) decreased the I_Ca_ 20 ± 5% (*n* = 5, *P* = 0.02); the co-application of 1 μM DAMGO additionally decreased the I_Ca_ 18 ± 7% for a total inhibition of 38 ± 7%, indicating that ω-ctx-GVIA occluded 51% of the DAMGO effect (*n* = 5, *P* = 0.03, ANOVA). To further characterize the Ca^2+^ channel subtypes involved in the DAMGO effect, an experimental series was performed in which the co-application of 3 μM ω-ctx-GVIA and 10 μM nifedipine inhibited 46 ± 6% of the I_Ca_, the addition of 1 μM DAMGO did not produce any further decrease of the remnant I_Ca_ (*n* = 6, *P* = 0.002, ANOVA) (Figures 4B,C). It is worth noting that the action of ω-ctx-GVIA is not reversible; thus, its actions remain during the entire experimental time course, and the effects of the three drugs are added sequentially. These results showed that MOR activation targets the L- and the N-type Ca^2+^ currents. Because the blockade of the L- and N-type currents fully occluded the DAMGO effect, no further selective Ca^2+^ antagonists (R- or P/Q-type) were tested.

### I_Ca_ inhibition is mediated by a Gα_i/o_

**Figure 5:**
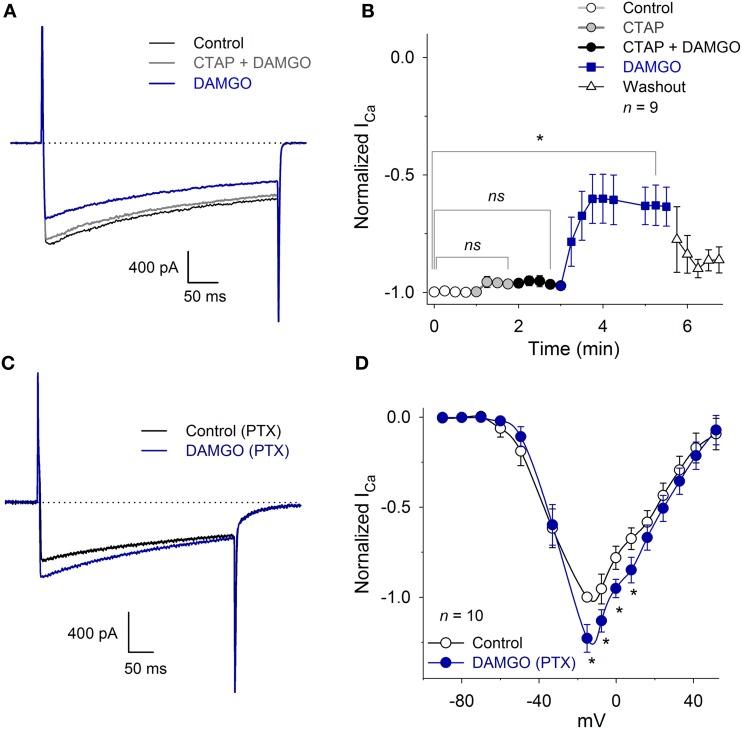
**CTAP and PTX occluded the inhibition of the I_Ca_ produced by MOR activation. (A)** Representative traces of the I_Ca_ under control conditions, 10 μM CTAP plus 1 μM DAMGO and 1 μM DAMGO. The CTAP had no action when applied alone, but occluded the inhibitory effect of DAMGO. In this and in the following graphs, the dotted line corresponds to zero current. **(B)** The perfusion of 10 μM CTAP did not modify the I_Ca_ amplitude. Co-application of CTAP and 1 μM DAMGO occluded the inhibitory effect of DAMGO, while the subsequent washout of CTAP allowed for DAMGO to inhibit the I_Ca_ 35 ± 8%; washout of DAMGO partially recovered the I_Ca_ (ns: *P* > 0.05, ^*^P < 0.05). **(C)** Traces of I_Ca_ in the control and with 1 μM DAMGO in a neuron incubated for 20 h with 500 ng/ml PTX. The use of DAMGO after incubation with PTX slightly increased the I_Ca_ (*P* = 0.015). **(D)** The I-V relationship (*V*_H_ = −100 mV) of the I_Ca_ in neurons incubated with PTX and after 1 μM DAMGO application. For voltages below −20 mV, DAMGO had no significant effect; for voltages between −20 to 20 mV, it increased the I_Ca_ (23 ± 7% at −15 mV) (^*^P < 0.05; *ns*: *P* > 0.05).

To determine whether the inhibition of the I_Ca_ was produced by the specific activation of the MOR, we studied the DAMGO effect in the presence of the MOR antagonist CTAP. The perfusion of 10 μM CTAP did not, by itself, modify the I_Ca_ amplitude (Figure 5B). Further, the co-application of 10 μM CTAP and 1 μM DAMGO did not reduce the I_Ca_ amplitude (CTAP + DAMGO 97 ± 1% with respect to the control, *n* = 9, *P* = 0.90). The CTAP washout, leaving only DAMGO, produced the expected decrease in the I_Ca_ peak amplitude (35 ± 8%, *n* = 9, *P* = 0.02, ANOVA; Figures 5A,B).

In an experimental series designed to demonstrate the participation of the Gα_i/o_ protein activation in the I_Ca_ inhibition produced by MOR activation, cultured VANs were incubated for 20 h with 500 ng/ml PTX. Afterwards, the perfusion of 1 μM DAMGO did not produce significant changes in the LVA current (*P* = 0.35). In contrast to the typical DAMGO inhibitory effects on the HVA component of the I_Ca_, DAMGO increased the HVA I_Ca_ 20 ± 7% (*n* = 10, *P* = 0.02) (Figures 5C,D). These results showed that the Gα_i/o_ protein is the transducer between MOR activation and LVA I_Ca_ inhibition; when the Gα_i/o_ protein is inhibited, there is evidence of the interaction between the MOR and other G proteins (see Discussion). No experiments were performed to characterize the signaling mechanism of the potentiation produced by DAMGO.

### I_Ca_ is not modulated by a voltage-dependent mechanism

**Figure 6:**
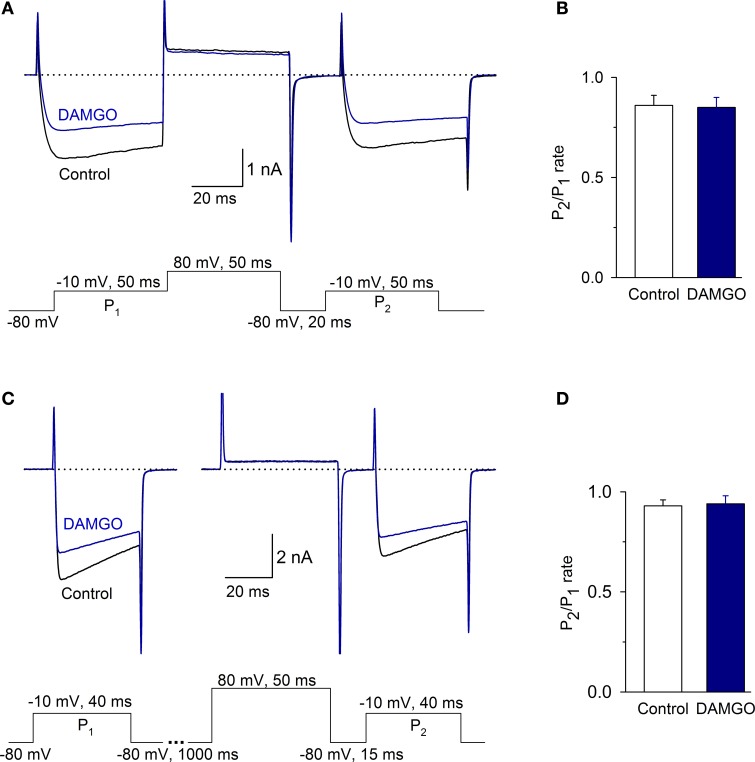
**The voltage-dependent signaling mechanism did not participate in the inhibition of the I_Ca_ by MOR activation. (A,B)** Traces of the I_Ca_ under the control condition and after 1 μM DAMGO perfusion. Protocols to evaluate the voltage-dependent modulation of the I_Ca_ consisted of two depolarizing pulses (*P*_1_ and *P*_2_, to −10 mV) separated by a depolarizing pulse that acted as a *P*_2_ prepulse (to 80 mV). **(B)** Bar graph showing the value of the *P*2/*P*1 ratio under control and after DAMGO. **(C)** The time between *P*_1_ and the pulse to 80 mV was 1 s to avoid inactivation accumulation. The ratio *P*_2_/*P*_1_ did not change between the control and after DAMGO application, independent of the time between *P*_1_ and the pulse to 80 mV. **(D)** Bar graph showing the value of the *P*2/*P*1 ratio under control and after DAMGO.

To determine whether MOR inhibition of I_Ca_ was voltage-dependent, we used a two-pulse protocol separated by a depolarizing pulse. Under control conditions, the *P*_2_/*P*_1_ ratio was 0.86 ± 0.05; with 1 μM DAMGO, the ratio was 0.85 ± 0.05 (the time between *P*_1_ and *P*_2_ was 70 ms, *n* = 6, *P* = 0.55, Figures 6A,B). To discard the possibility that there was an accumulation of channel inactivation (Ikeda, 1991), the *P*_2_/*P*_1_ ratio was also measured in experiments extending the interval between *P*_1_ and *P*_2_ to 1065 ms. In this condition, the *P*_2_/*P*_1_ ratio was 0.93 ± 0.03 in the control and 0.94 ± 0.04 after DAMGO perfusion (*n* = 8, *P* = 0.51) (Figures 6C,D). Finally, to further confirm whether Ca^2+^ channel facilitation was present in the VANs, the *P*_2_/*P*_1_ ratio with- and without depolarizing prepulses were evaluated; no significant differences were found between conditions, indicating that the channel facilitation was not present (data not show, *n* = 8, *P* = 0.86). These results show that in the isolated VANs, the voltage-dependent mechanism does not participate in the I_Ca_ inhibition by MOR.

### MOR effects on the HVA I_Ca_ are mediated by a cAMP pathway

To determine whether the voltage-independent signaling mechanism was involved in the I_Ca_ inhibition by MOR activation, we evaluated cAMP-pathway and PKC participation. We first list the results for the HVA and then for the LVA component.

**Figure 7:**
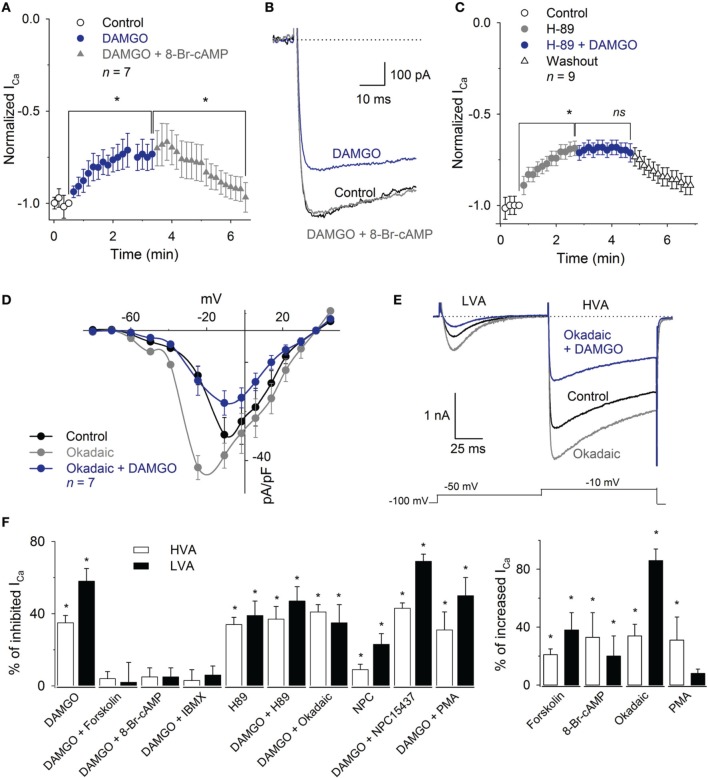
**Inhibition of the I_Ca_ by MOR activation was reverted by 8-Br-cAMP or IBMX and mimicked by H89. (A)** Time course of the I_Ca_ amplitude under the control condition, after 1 μM DAMGO and DAMGO plus 1 mM 8-Br-cAMP application. The application of 8-Br-cAMP reverted the DAMGO inhibition 92 ± 7% (*n* = 7). **(B)** Traces of the I_Ca_ under the control condition, after DAMGO and after the co-application of DAMGO and 8-Br-cAMP. **(C)** Time course of the I_Ca_ peak amplitude under the control condition, after the use of 1 μM H89 and after 1 μM H89 plus 1 μM DAMGO perfusion. The H89 mimicked the inhibitory effect of DAMGO; the co-application of H89 and DAMGO did not add to its inhibitory effects (*n* = 9). **(D)** I-V relationship of calcium current showed that the phosphatase inhibition with 100 nM okadaic acid enhanced the HVA and LVA currents, shifted the peak of the IV relationship to less depolarized values, but was unable to occlude the inhibitory effect of 1 μM DAMGO. **(E)** Representative traces of the LVA and HVA currents in control, under perfusion with 100 nM okadaic acid and coapplication of 100 nM okadaic acid and 1 μM DAMGO. The okadaic acid enhanced the I_Ca_ but was unable to occlude the DAMGO effect. The line dotted line represents zero current. The voltage clamp protocol is shown below the recordings. **(F)** Bar graph summarizing the effects produced by the various drugs used to discern the pathways involved in MOR actions upon both the LVA and HVA current components (^*^ means *P* < 0.05; ns means *P* > 0.05).

The application of the cAMP permeant analog 8-Br-cAMP (300 μM) enhanced the HVA I_Ca_ 33 ± 17% (*n* = 8, *P* = 0.02) and occluded the DAMGO inhibitory effect (*n* = 8, *P* = 0.25). Furthermore, 1 mM 8-Br-cAMP reverted the inhibition produced by 1 μM DAMGO on the I_Ca_ (recovered 92 ± 7% of the control HVA, *n* = 7, *P* = 0.87) (Figures 7A,B). The perfusion of the PKA inhibitor H-89 (1 μM) mimicked the effects produced by MOR activation, decreasing the HVA I_Ca_ 34 ± 4%; the co-application of 1 μM H-89 and 1 μM DAMGO did not added their inhibitory effects (*n* = 6, *P* = 0.81) (Figure 7C). The use of okadaic acid (100 nM) shifted the HVA activation curve to less depolarizing values (the V_1/2_ was −28 ± 2 mV under the control condition and −35 ± 3 mV with okadaic acid, *n* = 7, *P* = 0.04) and increased the maximum of the I_Ca_ I-V relationship 34 ± 8% (*n* = 7, *P* < 0.05). The subsequent co-application of okadaic acid and 1 μM DAMGO returned the V_1/2_ of the activation curve to the control values; it decreased the peak of the I-V relationship 51 ± 4% with respect to the okadaic acid application and 34 ± 4% with respect to the control (*n* = 7, *P* = 0.04 and *P* = 0.05, respectively, Figures 7D,E). The adenylyl cyclase activator forskolin (10 μM) enhanced the HVA I_Ca_ 21 ± 5% (*n* = 11, *P* = 0.03) and 10 μM forskolin prevented the 1 μM DAMGO inhibitory effect (*n* = 8, *P* = 0.33). The use of the phosphodiesterase inhibitor, 100 μM IBMX, reverted the inhibition of the I_Ca_ induced by 1 μM DAMGO (recovering 96 ± 6% of the I_Ca_, *n* = 6, *P*= 0.01, Figure 7F).

In contrast to the effects found with the cAMP-pathway related drugs, the activation or inhibition of PKC were not capable of modifying the MOR inhibition of the I_Ca_. The application of the PKC activator PMA (500 nM) enhanced the I_Ca_ 31 ± 16% (*n* = 5, *P* = 0.03). However, PMA perfusion was unable to recover the I_Ca_ from the inhibitory action of 1 μM DAMGO (*n* = 6, *P* = 0.78). The perfusion of the PKC inhibitor NPC15437 (200 nM) produced a significant inhibition of the I_Ca_ 12 ± 5% (*n* = 9, *P* = 0.01), while NPC15437 added its inhibitory effects with that produced by 1 μM DAMGO for a total inhibition of 42 ± 3% of the I_Ca_ (*n* = 9, *P* = 0.01, ANOVA) (Figure 7F).

### MOR effects on the LVA I_Ca_ are mediated by a cAMP pathway

With respect to the modulation of the LVA component, the use of 300 μM 8-Br-cAMP prevented the inhibition produced by 1 μM DAMGO (*n* = 5, *P* = 0.50, Figure 7F). The use of 100 nM okadaic acid enhanced the LVA 86 ± 8% (*n* = 5, *P* = 0.02), while the co-application of DAMGO (1 μM) and okadaic acid (1 μM) decreased the current 35 ± 10% with respect to the control (*n* = 5, *P* = 0.05). The use of forskolin (10 μM) produced an enhancement of the current (38 ± 12%, *n* = 8, *P* = 0.03), while the co-application of 10 μM forskolin plus 1 μM DAMGO occluded the DAMGO effect (*n* = 5, *P* = 0.18, Figure 7F). The co-application of 100 μM IBMX with 1 μM DAMGO, after DAMGO application, recovered the current to 93 ± 5% of the control (*n* = 5, *P* = 0.05). In addition, the inhibition of PKA with 1 μM H89 decreased the LVA current 40 ± 8% (*n* = 5, *P* = 0.02), whereas the subsequent co-application of 1 μM DAMGO did not change the remnant LVA current (*n* = 5, *P* = 0.26). Finally, the activation of PKC with 500 nM PMA did not significantly modify the LVA current or revert the DAMGO effect (*n* = 5, *P* = 0.95,). In contrast, the PKC inhibition with 200 nM NPC15437, after the DAMGO effect (41 ± 5%, *n* = 7, *P* = 0.01), added an additional inhibition of 23 ± 6% in the LVA current (*n* = 7, *P* = 0.05, Figure 7F). These results indicate that the LVA current is inhibited by MOR activation through the cAMP dependent signaling mechanism in a similar fashion to the mechanism mediating the HVA current inhibition.

### MOR activation effects on VAN action potentials

Taking into account the functional role of the I_K,Ca_ (Limón et al., 2005) and the potential role of the I_Ca_ in the setting of discharge patterns of VAN, the actions of MOR activation were also evaluated in current clamp conditions.

**Figure 8:**
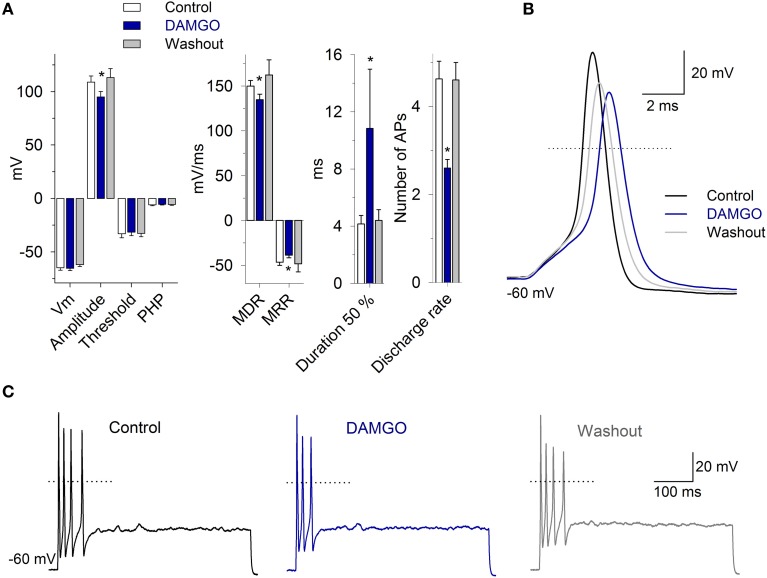
**The morphology and discharge of APs are modulated by MOR activation. (A)** Bar graph of the AP waveform parameters in the control, with 1 μM DAMGO and after drug washout (*n* = 11). DAMGO increased the AP duration and decreased the amplitude, the MDR and the MRR. **(B)** Representative AP recordings under the control condition, after DAMGO and the washout of the drug. **(C)** Recordings of cell response to depolarizing square current injection (400 pA, 500 ms). DAMGO (1 μM) decreased the discharge frequency of the cell. In **(B,C)**, the dotted line represents zero voltage. ^*^Indicates a significant difference *P* < 0.05.

To study the AP morphology, afferent neurons were stimulated with a 300 pA current pulse injection of 3 ms. The perfusion of 1 μM DAMGO decreased the AP amplitude from 109 ± 6 to 95 ± 5 mV, the MDR from 150 ± 6 to 135 ± 6 mV/ms, the MRR from −46 ± 3 to −39 ± 3 mV/ms, and increased the AP duration from 4 ± 0.5 to 10 ± 4 ms (*n* = 11, *P* < 0.05 for each parameter) (Figure 8). In 40% of the neurons studied, DAMGO blocked a rebound AP discharge after a hyperpolarizing pulse (200 pA during 500 ms). When the neurons were pretreated with *PTX* for 20 h, the perfusion of DAMGO (1 and 10 μM; *n* = 4 and *n* = 3, respectively) did not produce any significant change in the AP waveform (*P* > 0.05) or rebound discharge.

In the neurons that presented pulse-evoked repetitive discharge, DAMGO 1 μM decreased the number of APs in 73% of the neurons (11/15; from 4.7 ± 1 APs in the control condition to 2.6 ± 1 APs after DAMGO). This effect was completely reversible after drug washout (4.6 ± 1 APs, Figure 8) (*n* = 11, *P* = 0.03). In 3/15 neurons, the perfusion of DAMGO did not modify the discharge of the APs, and in 1 of the neurons, DAMGO application produced an enhancement in the AP discharge from 2 to 3 APs. The after hyperpolarization following the step depolarization did not change with the 1 μM DAMGO application, the control was −5.9 ± 4 mV and with DAMGO −5.7 ± 4 mV (*n* = 11, *P* = 0.49). These results suggest that the MOR activation did not modify the SK calcium activated potassium current.

**Figure 9:**
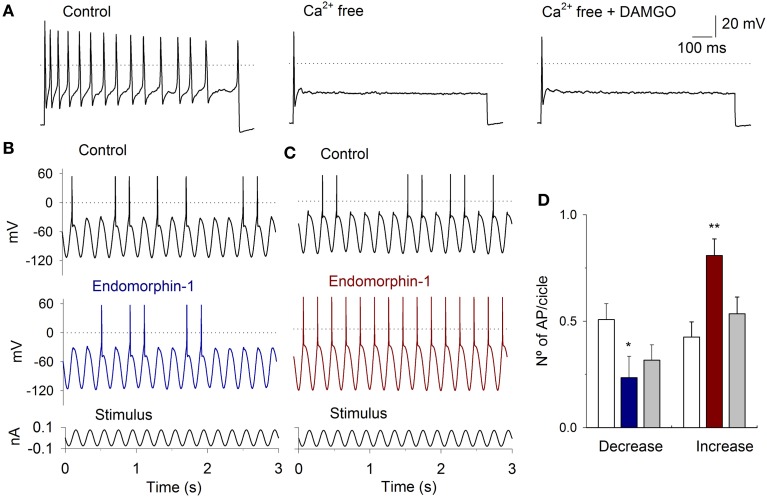
**Current clamp response to sinusoidal stimulation and MOR activation. (A)** A depolarizing current pulse injection (50 pA) produced a repetitive firing of the neuron The perfusion of VAN with Ca^2+^ free solution significantly decreased the AP discharge, the subsequent application od DAMGO did not further modified the AP discharge. **(B)** Typical response to sinusoidal current injection (5 Hz, 200 pA, shown below the record). Before the stimuli, the cells were held at −60 mV. In one group of cells, 1 μM endomorphin-1 decreased the APs per cycle. **(C)** In a second group of neurons, endomorphin-1 enhanced the number of APs per cycle. **(D)** Bar graph showing the mean number of APs per stimulus cycle in the control (white bars) and after MOR agonist application for the cells in which the MOR agonists decreased (blue) and increased (red) the AP discharge. Drug effect was partially reversible after washout of the drug (gray). (^*^means *P* < 0.05, ^**^means *P* < 0.01).

To further corroborate that the MOR activation modulates the I_Ca_ an experimental series with Ca^2+^ free extracellular solution was performed (Ca^2+^ was equimolarly substituted by Mg^2+^). With the perfusion of the Ca^2+^ free solution there were a decrement of the AP discharge (in 6 of 8 cells), in 2 cells, there was an enhancement of the AP discharge. The inhibitory or excitatory action was similar independently of the stimulus protocol (square pulses or sinusoidal stimulation). The use of 1 μM DAMGO in the Ca^2+^ free solution did not produce any significant change in the AP discharge in all the neurons studied (independently of their inhibitory or excitatory tendency after Ca^2+^ free perfusion), indicating that the MOR activity is due to the modulation of I_Ca_ (Figure 9A).

With the use of sinusoidal current stimulation of 5 Hz (stimulus amplitude selected to produce AP discharge in approximately 50% of the cycles), the use of MOR agonists 1 μM DAMGO (*n* = 6), 1 μM endomorphin-1 (*n* = 5) or 1 μM Met-enkephalin (*n* = 6) produced similar effects. In one group of neurons, MOR agonists decreased the AP discharge per cycle from 0.51 ± 0.07 to 0.23 ± 0.10 (*n* = 7, *P* = 0.007), while in the other group of neurons, MOR agonists increased the number of APs per cycle from 0.43 ± 0.07 to 0.81 ± 0.08 (*n* = 10, *P* = 0.004) (Figures 9B–D). The C_m_ of the neurons in which DAMGO agonists decreased the discharge was larger (33 ± 4 pF) than those in which an enhancement of activity was observed (23 ± 3 pF; *P* = 0.02).

## Discussion

We found that all VANs express the HVA I_Ca_ and that 68% of the neurons also express the LVA I_Ca_. The neurons that lacked the LVA current were significantly smaller than the neurons with both LVA and HVA currents. The percentage of neurons with LVA current is comparable (although slightly smaller) to previously reported values of 74% (Limón et al., 2005) and 81% (Desmadryl et al., 1997). The difference, although small, may be due to the larger sample size in this study (187 neurons) and to differences in the species studied (Wistar rats P7–10, Swiss rats P4-P8, and, in our case, Long Evans P7–10).

The VANs express voltage gated calcium channels of the T-, L-, N-, P/Q- and R- types (Desmadryl et al., 1997; Chambard et al., 1999; Autret et al., 2005; Limón et al., 2005; Meredith et al., 2011). The modulation of the HVA and LVA I_Ca_ currents influence the VAN excitability, thus modulating the vestibular output; our results showed that the T-, L- and N-type channels are modulated by MOR. In the VANs, the N-type current may be mostly related to neurotransmitter release at the vestibular nucleus neurons. The L-type current may participate in the calcium transients in the cell body and dendrites of the vestibular neurons (Meredith et al., 2011). Further, the L-type current may modulate gene expression (Catterall, 2011). The T-type current may affect the synaptic integration of calyx endings and also contribute to the differences between the regular and irregular discharge of the VANs (Autret et al., 2005). This suggests that the MOR may participate in regulating the gain of the afferent neurons contributing to maintain the strict output balance from the vestibular endorgans.

The IC_50_ of the DAMGO effect in our experiments was in the nanomolar range, which is similar to that reported in other isolated primary neurons, such as the nodose superior cervical or trigeminal ganglion neurons (Rusin and Moises, 1998; Margas et al., 2007). The DAMGO effect did not affect the voltage dependence or the activation voltage of the I_Ca_, indicating that the inhibition was due to a decrease in the channel open probability and not to a modification of channel kinetic properties.

Both the HVA and LVA Ca^2+^ currents were modulated by MOR in the VANs. The HVA modulation by MOR has been found in several neuronal types (Rusin and Moises, 1998; Connor et al., 1999; Lemos et al., 2012) and in heterologous expression systems (Bourinet et al., 1996; Margas et al., 2007). However, there are only a few reports that have shown a modulation of the LVA current by MOR activation (Schroeder et al., 1991; Formenti et al., 1995; Yang et al., 2000). The magnitude of HVA modulation by MOR varies in different neuronal types. In our case, the inhibition (35%) was similar to that found in the periaqueductal gray neurons (Connor et al., 1999) and nodose ganglion neurons (Rusin and Moises, 1998) and was significantly lower than the 64% inhibition reported in superior cervical ganglion neurons (Margas et al., 2007); these differences may be due to the subtypes of HVA Ca^2+^ channels involved or to intracellular signaling mechanism specificities. In the case of the LVA I_Ca_, the percentage of inhibition in the VANs by MOR activation was 58% in the P7–10 rats. This is larger than data previously reported for the modulation of the LVA by MOR activation in dorsal root ganglion neurons of the rat (≅ 35%), thalamic relay neurons (40%) and neuroblastoma cells N1E115 (≅ 20%) (Schroeder et al., 1991; Formenti et al., 1995; Yang et al., 2000). The effects of MOR activation in P28–30 rats in our experiments (36%) were closer to the values reported in the mentioned systems.

In our experiments, the action of DAMGO was prevented by the specific MOR antagonist CTAP, suggesting that the DAMGO effect is specifically due to MOR activation (Abbruscato et al., 1997; Margas et al., 2007). In addition, the inhibition of the Gα_i/o_ protein with PTX prevented the inhibitory effects on the LVA and HVA Ca^2+^ current components. It is noteworthy that the Gα_i/o_ inhibition by PTX inverted the action of MOR activation in the HVA Ca^2+^ current; thus, DAMGO application produced an enhancement of the HVA I_Ca_. This positive modulation of the HVA I_Ca_ may be due to the interaction of the opioid receptor with Gα proteins other than Gα_i/o_ (Cruciani et al., 1993) or as consequence of the modulation by Gβγ of the calcium channels (Smrcka, 2008). While the Gα_s_ may be involved in the enhancement of the HVA I_Ca_, there are no specific antagonists of this G-protein. Further, the use of GDP-β-S, which is an unspecific G protein inhibitor, implies rupturing the cell patch; this produces a condition in which the I_Ca_ has a significant rundown that very quickly reduces the experimental reliability, thus making it useless. In further examination of the signaling mechanism involved in the I_Ca_ modulation by MOR, we found that the VANs Ca^2+^ channels were in a non-saturated level of phosphorylation susceptible to either positive or negative modulation in our experimental conditions.

The signaling mechanism involved in the I_Ca_ modulation by MOR may be voltage-dependent (VD) or voltage- independent (VI) (Law, 2011). In the VANs, the VD mechanism did not participate in the I_Ca_ inhibition by MOR, similar to what has been found in the globus pallidus neurons (Stefani et al., 2001). The lack of VD modulation in the VANs may be due to various factors: (i) the Gβγ may be a type that cannot interact with the AID site (Smrcka, 2008), (ii) the Gα_i/o_ may also interact with the AID site instead of the Gβγ subunit (Kinoshita et al., 2001), (iii) the AID site may be phosphorylated by PKC, impeding the Gβγ interaction (Zamponi and Snutch, 1998), and (iv) the β subunit may compete with the Gβγ complex for the AID site (Bourinet et al., 1996). The evaluation of the cAMP pathway shows that in the VANs, the HVA and LVA I_Ca_ inhibition produced by MOR activation occurs through the VI mechanism; this finding is consistent with previous results reported in the literature (Law, 2011).

**Figure 10:**
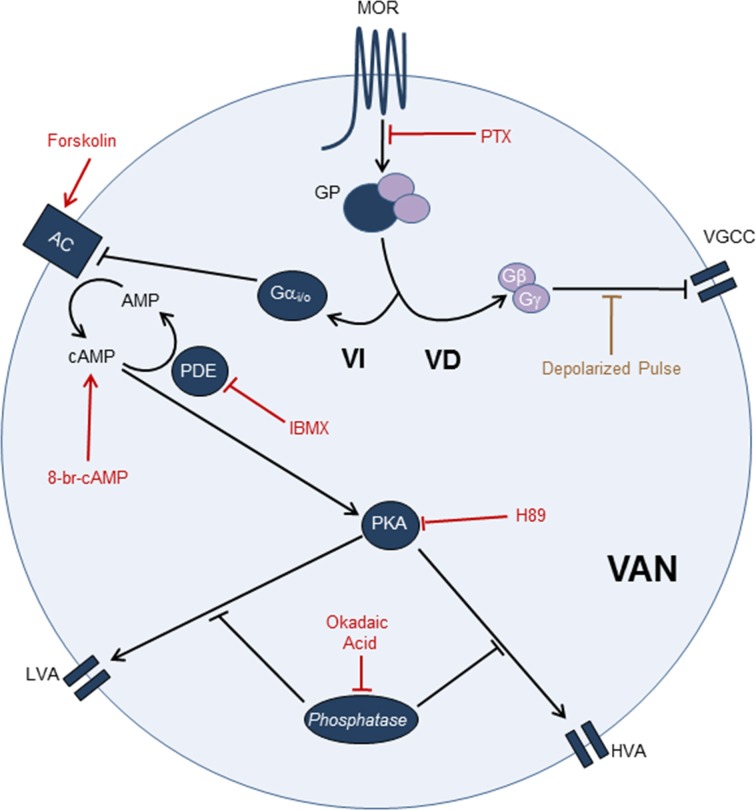
**MOR activation pathway leading to I_Ca_ inhibition in the VANs.** The use of PTX showed that the I_Ca_ inhibition by MOR activation is Gα_i/o_ dependent. Use of paired pulses showed that the VD signaling mechanism does not significantly participate in the I_Ca_ inhibition. The use of forskolin, 8-Br-cAMP, IBMX, H89, and okadaic acid indicate that the I_Ca_ inhibition (LVA and HVA) by MOR activation is mediated by the cAMP pathway. Involving the Gα_i/o_ inhibition, AC inhibition, decreased of cAMP levels, decreased of PKA activity and decreased of PKA-dependent phosphorylation of calcium channels. In the scheme, arrow-endings indicate activation and the line-endings indicate inhibition. The names and lines in red indicate the drugs used. AC, Adenylyl cyclase; GP, G Protein; PDE, Phosphodiesterase; VD, Voltage dependent signaling mechanism; VGCC, Voltage gated calcium channels; VI, Voltage independent signaling mechanism.

Thus, the MOR modulatory pathway for the I_Ca_ inhibition in the VANs involved a Gα_i/o_ coupled mechanism that, by decreasing the AC activity, led to a reduction in the cAMP levels and consequently decreased the PKA activity; these effects may ultimately diminish the calcium channel phosphorylation and the channel open probability (Figure 10). Our results also suggest that the MOR can also interact with other G proteins, such as Gα_q_ and Gα_s_, with no participation of the PKC as a second messenger.

In current clamp experiments, it was thought that the decrease in the action potential amplitude and the MDR would be caused by a decrease in the sodium current (I_Na_). However, this idea is not supported by the experimental data because the fast-inactivating inward-current in the total current voltage-clamp experiments (that is almost completely I_Na_) showed little or no inhibition by MOR activation. The VANs have a LVA Ca^2+^ window current between −65 and −45 mV (Autret et al., 2005); thus, the LVA participate in the depolarization upstroke of the AP suggesting that changes in the amplitude, and the MDR may be caused by the inhibition of the LVA component. The change in the MRR and duration may be secondary to an inhibition of the calcium dependent potassium current (I_K,Ca_) (Limón et al., 2005). In VANs with square-pulse-evoked repetitive discharge, MOR activation inhibited the discharge in 73% of the cells and enhanced it in only one neuron. Using sinusoidal stimulation and MOR agonist application, two types of responses were observed: one excitatory and the other inhibitory, which indicate that MOR may exert a complex modulation of the VANs depending on the expression of currents such as the I_Ca_ and the I_K,Ca_ (Limón et al., 2005; Lysakowski et al., 2011; Meredith et al., 2011). In those neurons with a higher density of I_K,Ca_, the inhibition of the I_Ca_ led to an increase in excitability because there was a net decrease of outward current. In contrast, in those neurons with a low density of the I_K,Ca_, the inhibition of the I_Ca_ predominantly produced a decrease in the inward current, thus leading to a decrease in excitability. However, we must consider that MOR and membrane ionic currents of the VAN are coupled, thereby forming a dynamic network; thus, changes in any of the currents could result in changes in the activation and eventually may also alter the dynamics of other currents.

In the central synapse of the VANs within the vestibular nucleus neurons, the I_Ca_ inhibition mediated by the MOR could significantly modulate the vestibular nucleus neuron gain, thus significantly contributing to the mechanisms proposed to participate in the discrimination of the voluntary head movements (Cullen, 2012). In addition, in the vestibular compensation produced after an unilateral labyrentectomy, the inhibition of the I_Ca_ may contribute to vestibular plasticity through the modulation of gene expression (Kitahara et al., 2006; Catterall, 2011; Beraneck and Idoux, 2012). The accuracy of the vestibular system critically depends on the balance between the gains of both vestibules (Sadeghi et al., 2009; Cullen, 2012). For this to be achieved, complex mechanisms (formed by the neuronal network and modulator molecules) able to regulate the network gains are required. The MOR may act by modulating the VAN operational range; modifying the postsynaptic signal integration, excitability and action potential discharge; and the neurotransmitter release in the afferent synapse at the vestibular nucleus.

### Conflict of interest statement

The authors declare that the research was conducted in the absence of any commercial or financial relationships that could be construed as a potential conflict of interest.
